# Supplementary material for: Patterns of Intron Gain and Loss in Fungi
Source: PLoS Biol. 2004 Nov 30;2(12):e422. doi: 10.1371/journal.pbio.0020422 (PMC532390; doi:10.1371/journal.pbio.0020422)
Supplement: Table S1 — Also available at http://genes.mit.edu/NielsenEtAl/. (4.3 MB ZIP). [file pbio.0020422.st001.zip › NielsenEtAl/html/242.html]

AN6145.1.NCU08554.1.MG07389.1.FG06532.1


```
 CLUSTAL W (1.82) Multiple Sequence Alignments - Introns Inserted


Sequence 1: AN6145.1	210 aa
Sequence 2: FG06532.1	177 aa
Sequence 3: NCU08554.1	186 aa
Sequence 4: MG07389.1	176 aa
Alignment Length: 213 aa
Number Identitical Residues: 97 aa
Alignment Score (without introns) 4437


MG07389.1 	-MADQ0H~PETGLPPNWEVRLSKSKNLPYYFNTSNTTSRWEPPPGTDTDKLKQYMGKYHS
NCU08554.1	----M0T~IETGLPEDWEVRHSQSKNLPYYFNSATKTSRWEPPSGTDVDKLKIYMAKYHS
FG06532.1 	-----~M0ADTGLPPGWEVRHSNSKNLPYYFNSAEKLSRWEPPSGTDTEKLKHYMATNHS
AN6145.1  	MIFRQ~K~VNTGLPAGWEVRHSNSKNLPYYFNPATRESRWEPPADTDMETLKMYMATYHS
          	         :**** .**** *:*********.:   ******..** :.** **.. **

MG07389.1 	SALPSDSPQ-----AGQIRAAHLLIKHRDSRRPSSWKE~ENITRTKEDARRIIEAHKDRI
NCU08554.1	PTSQQQQQQQQQQPQGKIRCAHLLVKHNQSRRPSSWRE0SEITRTKQEALTTLQGFEQRI
FG06532.1 	AGSRPGAVPG--VPEGKIRAAHLLVKHRDSRRPSSWRE0AEITRTKEEAFEIIKEHEQKI
AN6145.1  	GAATYHEAP---SQEGKIRCSHLLVKHRDSRRPSSWRE~AEITRTKEEAREILRGHQERI
          	               *:**.:***:**.:*******:*  :*****::*   :. .:::*

MG07389.1 	ASGEASLAALATTESDCSSARKRGDL~GFFGRGDMQKEFEEAAFALKPGEISGVVDTASG
NCU08554.1	KSGSISLGELALTESDCSSARKRGDL~GYFGRGDMQKEFEDAAFALKPGEISGIVDTASG
FG06532.1 	KSGSVSLGELALTESDCSSARKRGDL~GYFGRGDMQKEFEDASFGLSPGQMSEIVETASG
AN6145.1  	MRGEIRLGDLAMSESDCSSARKKGDL2GFFGRGEMQKEFEEAAFALQPGQVSDIVESGSG
          	  *.  *. ** :*********:*** *:****:******:*:*.*.**::* :*::.**

MG07389.1 	LHLIER~-------------------------------
NCU08554.1	LHLIER2RWQDGHHS-----------------------
FG06532.1 	LHLIER2LE-----------------------------
AN6145.1  	LHLIER~YVSFSMPWVALIMLFFTGYNRYCWALLFWYI
          	******   . .    :      :. .  . :
```
